# Anxiety and depression: A top-down, bottom-up model of circuit function

**DOI:** 10.1111/nyas.14997

**Published:** 2023-05-02

**Authors:** Deryn O. LeDuke, Matilde Borio, Raymundo Miranda, Kay M. Tye

**Affiliations:** 1 Salk Institute for Biological Studies, La Jolla, California, USA; 2 Biomedical Sciences Graduate Program, University of California San Diego, La Jolla, California, USA; 3 Neurosciences Graduate Program, University of California San Diego, La Jolla, California, USA; 4 Howard Hughes Medical Institute, La Jolla, California, USA; 5 Kavli Institute for the Brain and Mind, La Jolla, California, USA

**Keywords:** amygdala, anxiety, attractor states, bottom-up, top-down, depression, PFC

## Abstract

A functional interplay of bottom-up and top-down processing allows an individual to appropriately respond to the dynamic environment around them. These processing modalities can be represented as attractor states using a dynamical systems model of the brain. The transition probability to move from one attractor state to another is dependent on the stability, depth, neuromodulatory tone, and tonic changes in plasticity. However, how does the relationship between these states change in disease states, such as anxiety or depression? We describe bottom-up and top-down processing from Marr’s computational-algorithmic-implementation perspective to understand depressive and anxious disease states. We illustrate examples of bottom-up processing as basolateral amygdala signaling and projections and top-down processing as medial prefrontal cortex internal signaling and projections. Understanding these internal processing dynamics can help us better model the multifaceted elements of anxiety and depression.

## INTRODUCTION

According to the most recent National Institute of Mental Health reports on US adults, anxiety and depression have a lifetime prevalence of 30% and 20%, respectively.^1,2^ Treatment options remain unsatisfactory for a significant number of patients struggling with the disorders, due in part to a lack of understanding of the underlying mechanisms.^3^ In the last decades, neuroscience research has made important advances in gaining a better understanding of the mechanisms that could give rise to such disorders.

In this review, we provide an overview of the literature to answer three central questions about disease states in the brain:

1. What accounts for the brain’s ability to shift from a healthy to a diseased state?
2. How does this shift affect how stimuli are processed?
3. Which physical neural-dynamic changes drive this shift?

**FIGURE 1:**
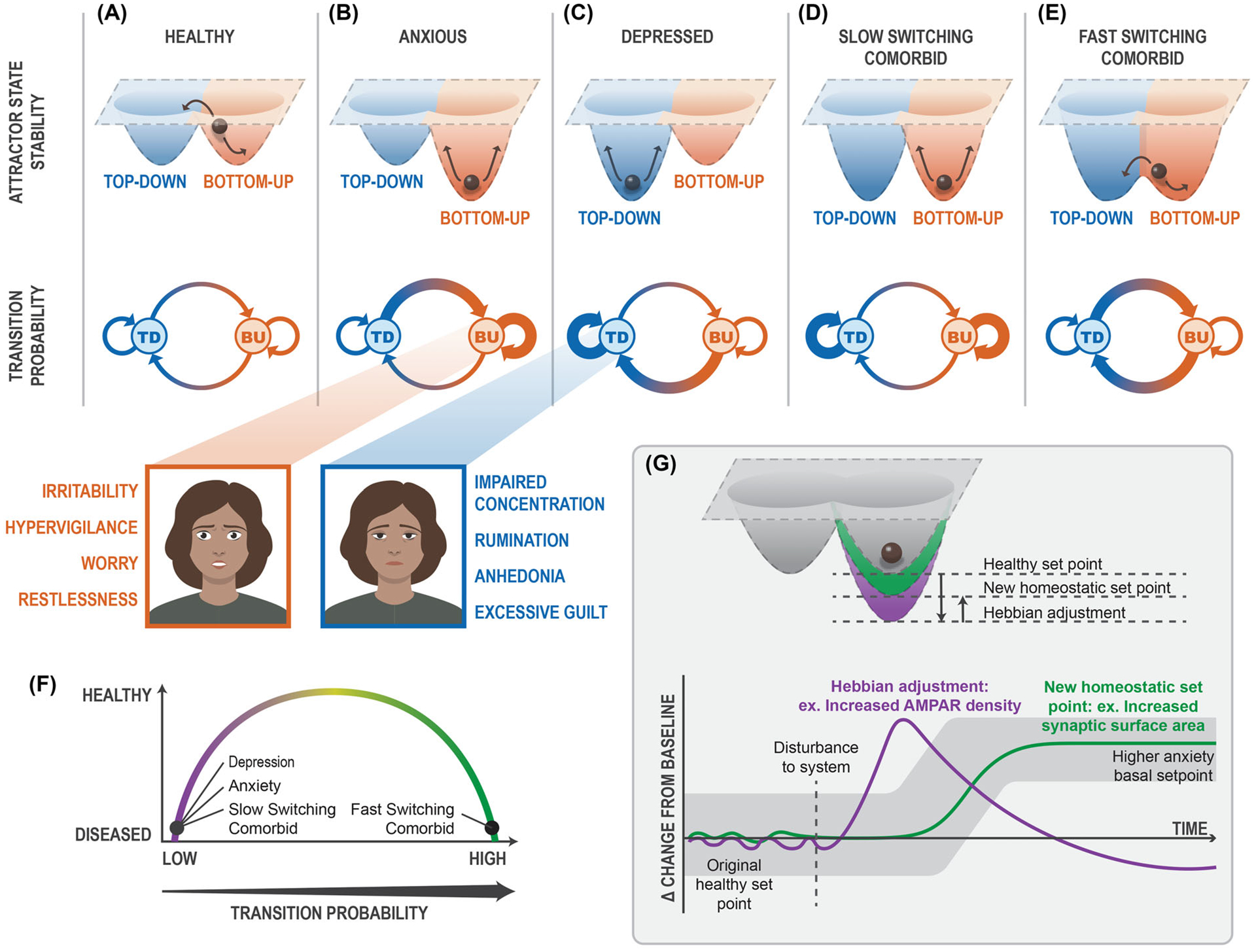
Attractor state dynamics of bottom-up, top-down processing. Bottom-up (orange) and top-down (blue) processing in (A) healthy, (B) anxious, (C) depressed, (D) slow-switching comorbid, and (E) fast-switching comorbid states are represented through attractor state dynamics (upper), where the ball indicates population activity at a given state with arrows showing average range of motion, and through the transition probability between states (lower), where the thicker arrows indicate higher transition probabilities. (F) Disordered transition probabilities result in different phenotype presentations. When the transition probability between TD/BU states is critically low, patients present anxiety, depression, or slow-switching comorbidity. When the transition probability between TD/BU states is critically high, patients present fast-switching comorbidity. (G, upper) Attractor state depth can change from Hebbian (purple) and homeostatic (green) plasticity changes. (G, lower) Plasticity changes are altered on different timescales; whereas Hebbian plasticity deviates from the basal setpoint, homeostatic plasticity resolves plastic deviations and, if needed, re-establishes the basal setpoint. Abbreviations: AMPAR, α-amino-3-hydroxy-5-methyl-4-isoxazolepropionic receptor; BU, bottom-up; TD, top-down.

**FIGURE 2:**
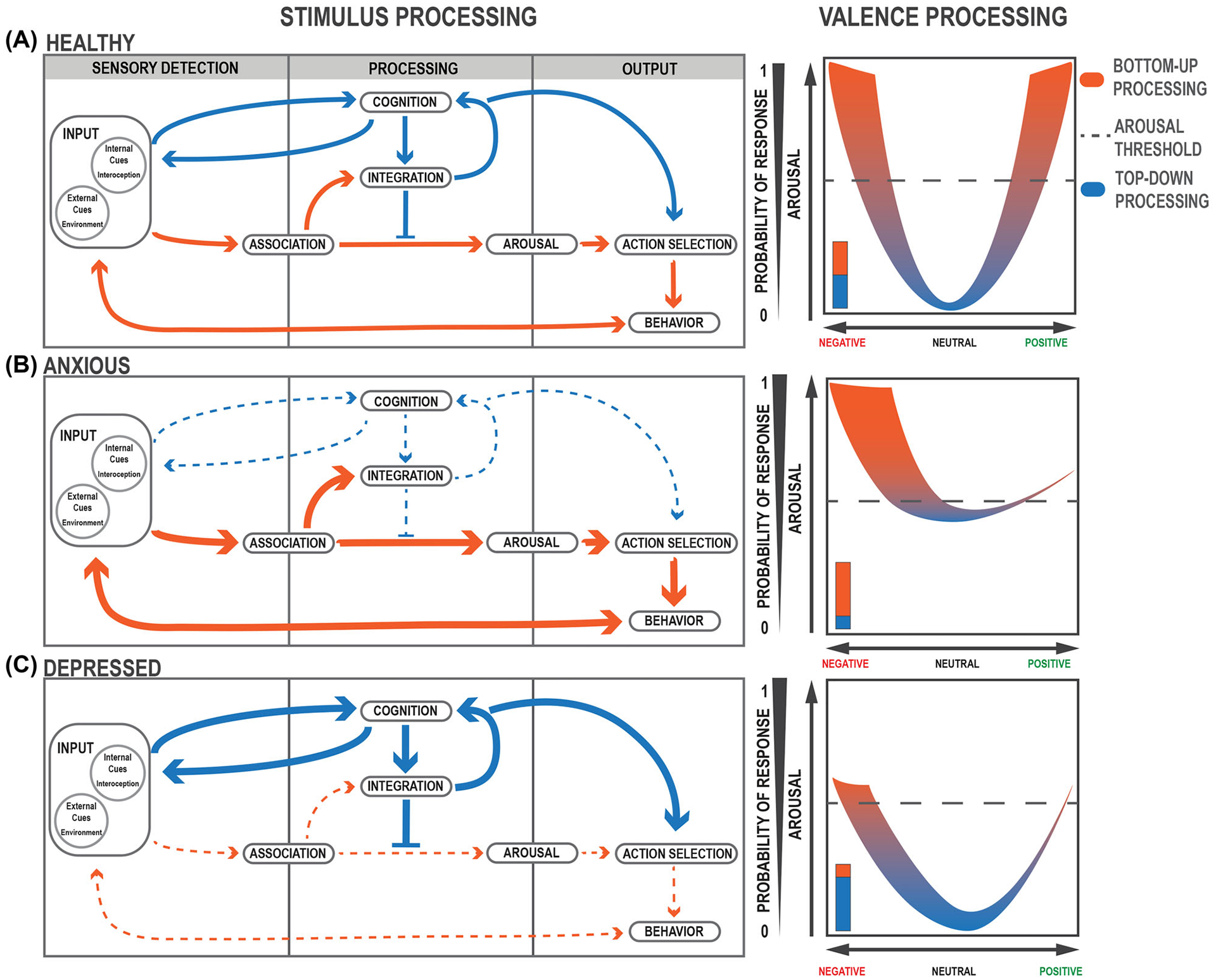
Interaction of bottom-up and top-down feedback loops during stimulus processing. (A–C, left) Flow of stimulus and valence processing in (A, left) healthy, (B, left) anxious, and (C, left) depressed states. Larger, thicker arrows indicate increased bias or activation. Dashed, thinner arrows indicate decreased activation. (A–C, right) Valence processing curves. (A, right) In a healthy state, valence processing corresponds appropriately to increases in arousal and positive–negative valuations of valence. The dashed line indicates the threshold of responding to a given stimulus. (B, right) In an anxious state, arousal is increased with a negative valence bias. (C, right) In a depressed state, arousal is decreased with a negative valence bias.

**FIGURE 3:**
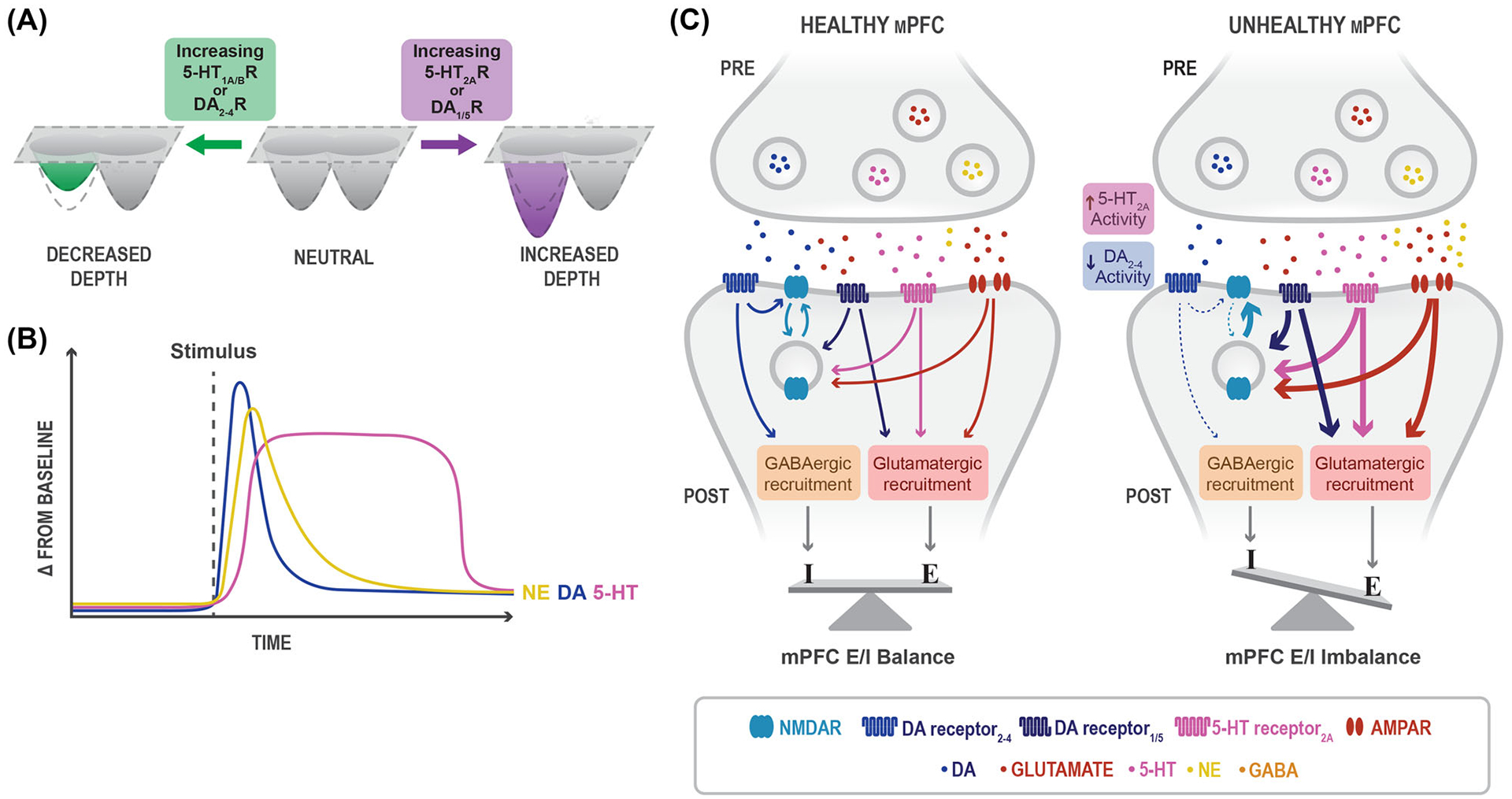
The effects of neuromodulation on the depth of attractor states and synaptic plasticity. (A) As neural populations receive higher bouts of 5-HT_1A/B_ receptor activity and/or increased DA_2–4_ receptor activity, the depth of an attractor state decreases. As neural populations experience higher levels of DA_1/5_ receptor activity and/or increased levels of 5-HT_2A_ receptor activity, the depth of an attractor state increases. (B) Variable release of neuromodulation across time after receiving a given stimulus. DA (blue) and NE (yellow) exhibit short encoding of stimulus. 5-HT (pink) imposes a slower, sustained release onto synapses. (C) In healthy mPFC synapses, 5-HT_2A_ receptors (pink), D1-like receptors (dark blue), and AMPARs (red) interact to encourage NMDAR externalization. These receptors additionally recruit glutamatergic neurons (red). D2-like receptors (blue) internalize surface NMDARs and recruit GABAergic neurons (orange). Under unhealthy conditions in the mPFC, increased pressure from 5-HT_2A_ and D2-like receptors (in addition to decreased pressure from D1-like receptors) causes increased externalization of NMDARs and glutamatergic neuron recruitment. The consequence of this synaptic change is an excitatory/inhibitory imbalance in the mPFC. Abbreviations: 5-HT, serotonin; AMPAR, α-amino-3-hydroxy-5-methyl-4-isoxazolepropionic receptor; DA, dopamine, E, excitation; GABA, gamma-aminobutyric acid; I, inhibition; mPFC, medial prefrontal cortex; NE, norepinephrine; NMDAR, N-methyl-D-aspartate receptor; POST, postsynaptic neuron; PRE, presynaptic neuron.

To address these questions, we draw from David Marr’s analytical model to propose a three-level computational-algorithmic-implementational framework centered around the hypothesis that shifts in *top-down* (TD) and *bottom-up* (BU) processing occur to produce anxiety and depression as distinct disease states.^4^ First, we describe how changes in the computational landscape (attractor state dynamics) account for these shifts in processing (Figure 1). Then, at the algorithmic level, we describe how stimulus and valence processing maintain these states and become disrupted (Figure 2). Lastly, we identify and summarize findings on key brain structures, including the amygdala and the prefrontal cortex (PFC) as representative centers of BU and TD processing, respectively, which at the circuitry level contribute to the biological implementation of these changes (Figure 3). Integrating research within each of these fields can allow us to come to a deeper understanding of mental health disorders and provide a path toward more efficacious treatments. Anxiety and depression are complex diagnoses; manifestations of anxious and depressive pathologies are diverse, as reflected in the diagnostic criteria specified in the most recent edition of the Diagnostic and Statistical Manual of Mental Disorders (DSM-V). To define anxiety and depression as we discuss them in this review, we outline common symptomatic criteria delineated in the DSM-V, categorized by psychosocial and physiological deficits (Box 1).^5^ Other reviews on anxiety and depression discuss the dysfunction of dopaminergic valence circuits,^6,7^ the biological variable of sex,^8^ and the dysfunction of threat-related circuitry.^9,10^ It is worth noting that multifaceted disorders require multifaceted frameworks and thus we endeavor in our model to incorporate the collective computational, psychodynamic, and biological investigations into these illnesses (see Box 1).

## ATTRACTOR STATES ARE REPRESENTATIVE OF NEURAL NETWORK ACTIVITY

The brain is a dynamic system that depends on multiple hidden variables, of which we can only observe a minority. The ongoing activity of a single cell is governed by the combination of inputs: their amplitude, location, timing, and regulation of ion channels combined with slower signals from neuromodulation. On a larger scale, a cell receives inputs from tens of thousands of other neurons, whose connections and activity are unknown. Such a vast number of hidden variables in neural activity demand computational models that can represent biological activity accurately—understanding the brain as a dynamic system accounts for these hidden variables that cannot be observed.^11–13^ Such models contain “attractor states,” which represent states that a system gravitates toward regardless of initial conditions.^11^ As Antonio Damasio argues in his work, *The feeling of what happens: Body and emotion in the making of consciousness*, motivational senses of self emanate from a *proto-self—*a collection of patterns that summate and map the momentary homeostatic needs of an individual in many dimensions.^14^ Attractor states manifest as the computational foundations of the brain, upon which we construct our psychosocial identity. Attractor states are often likened to a bowl: if network activity was a ball, the ball would gravitate toward the deepest, or most stable, part of the bowl. Moreover, the deeper the bowl, the more energy it would require for the ball to roll out. This concept from mathematical systems can also be applied to biological systems, and this framework has been used in the context of mental health and disease.^12,15–18^ To advance our understanding of neural dynamics in the context of mental health, viewing the brain as an attractor state network allows us to make predictions based on past activity.

Mounting evidence suggests that neural ensembles demonstrate activity that is similar to attractor state models.^11,18–22^ An attractor network (distinct from the attractor *state*) is a type of dynamic network that evolves toward stability over time. To return to the bowl, an attractor state creates a malleable bowl, which can be deepened or otherwise augmented with significant, persistent changes in activity.^23^ For example, foam can be easily depressed with force, while removing your hand and pushing down on another area will change the location and depth of the depression. Many networks with continuous dynamics will develop attractor states that minimize energy in the system and are thus hyperstable.^11,13,24^ Population activity can and will deviate from an attractor state while processing different stimuli (noise in the network system); however, network activity will ultimately converge back to a stable state. The minimum level of energy in a system at which network activity converges is known as the *basin of attraction*.^23,25^ In neural populations with strong excitatory connections, multiple stable states can exist, all with basins of attraction.^25^ To classify the relative stability of mental states, we can use an attractor network to model the brain’s ability to transition between different forms of processing. Attractor states represent the first, computational level in our framework, in which the transition probabilities of network activity influence biological and behavioral phenotypes. We illustrate a multipoint attractor manifold to focus on specific components of anxious/depressive disease states in the brain. This framework does not invalidate the possibility for other attractor networks and their potential influence on network activity.

Patients with diagnosed anxiety and depression reveal different brain states as represented by functional MRI (fMRI) readouts, suggesting that anxiety and depression exist in separate attractor states.^26^ When comparing the response to sad facial expressions in patients with major depressive disorder (MDD) and generalized anxiety disorder, PFC activity is positively correlated with depressive symptoms, whereas it is negatively correlated with anxious symptoms. Human imaging studies indicate a significant relationship between anxious arousal and increased amygdalar–subcortical activity. In humans experiencing anhedonia, researchers observe increased limbic–paralimbic activity.^26–28^ Interestingly, areas with increased activity in anxiety had decreased activity in patients with depression and areas with decreased activity in anxiety had increased activity in patients with depression. The existence of these separate brain states supports the theory that there are bistable attractor states that can represent anxiety and depression (Figure 1A–E). As individuals exhibit an increased tendency toward TD or BU processing, attractor states reflect a similar geometry—the depth of one attractor state represents the increased likelihood that network activity will gravitate toward that type of processing.^12^ As we illustrate in Figure 1, healthy individuals can smoothly alternate between TD and BU processing states to modulate behavior appropriately. In pathologic conditions, the depth of the attractor states increases such that network activity is heavily biased toward either TD (in depression) or BU (in anxiety) (Figure 1B,C).

The concurrence of both anxiety and depression (comorbid anxious-depression) is very common, occurring in roughly half of cases and presenting an even greater challenge for treatment.^2,29–31^ It is possible that when the energy dynamics of one attractor state is disrupted, other states can be either compensatory or decompensatory. In our model, both attractor states are hyperstable in comorbid anxious-depression, leading to concentrated switching between pathological levels of TD and BU processing (Figure 1D–F). However, maladaptive stability may occur with different transition probabilities (Figure 1F). These differences in transition probability are termed in this review as a “slow-switching” comorbid state, where a person may be diagnosed with anxiety and depression over the course of their lifetime (Figure 1D), and “fast-switching” comorbid state, wherein a rapid state leads an individual to perceive symptoms of anxiety and depression simultaneously (Figure 1E).

## PLASTICITY MODIFIES THE ARCHITECTURE OF ATTRACTOR STATES

Changes in the geometry of an attractor state may represent neural dynamic changes in firing rate or synaptic strength. Increased depth of attractor states has been biologically described by excitatory glutamatergic ion receptor function and spine concentration (Figure 1G).^12^ Experimental evidence demonstrates that reduced N-methyl-D-aspartate receptor (NMDAR)/α-amino-3-hydroxy-5-methyl-4-isoxazolepropionic receptor (AMPAR) function in a given brain region influences processing capabilities, such as in associative learning and memory.^32^ To achieve an attractor state, neurons must retain a level of stability while being flexible enough to receive and transmit new information. This dynamic version of stability is achieved by neural plasticity, mechanisms by which neurons change in sensitivity and strength. Neural plasticity is generally described as two opposing mechanisms, Hebbian and homeostatic plasticity.^33^ Hebbian plasticity deviates neural activity from homeostasis and homeostatic plasticity encourages neural activity back to a predetermined setpoint^34^ (Figure 1G). These two types of plasticity happen on different timescales, Hebbian plasticity changes neurons acutely, whereas homeostatic plasticity occurs over a longer timescale (Figure 1G). Hebbian plasticity occurs when NMDARs in the synapse are activated, resulting in a signaling cascade wherein AMPAR density increases and the synapse becomes more sensitive to stimulation.^35^ Homeostatic plasticity is a form of metaplasticity, which affects neurons’ ability to undergo further synaptic changes based on previous plasticity. Homeostatic plasticity can consist of scaling receptors to different synapses, which in turn alters the sensitivity of the neuron.^33^ In the context of attractor networks, plasticity is the way that attractor states can change and stabilize their geometry. While the depth of attractor states is dependent on plasticity, changes in plasticity probability can be induced through long-term enhancement or depression of excitability via homeostatic plasticity.^36^

The mechanisms of Hebbian and homeostatic plasticity biologically influence the strength and identity of further plasticity.^37^ Hebbian plasticity modifies synaptic strength in the same direction of a given stimulus. For example, strong stimulation leads to an increase in synaptic strength resulting in long-term potentiation (LTP); conversely, low-frequency stimulation weakens synaptic strength and causes long-term depression.^38,39^ Homeostatic plasticity operates in contrast to Hebbian plasticity, such that the threshold for further Hebbian plasticity is modified. Because homeostatic plasticity intends to maintain a homeostatic setpoint for a neural network, it utilizes biological mechanisms to (A) *acutely* return activity to a baseline and (B) *chronically* modify a baseline to adapt to periods of high activity. As we illustrate in Figure 1G, a period of activity deviating from baseline increases the threshold for induction of LTP by methods of synaptic scaling or genetic augmentation of neuronal activity.^34,37^ The conclusion of homeostatic plasticity is thus a modification of thresholds for Hebbian plasticity and maintenance of a new setpoint following a period of high activity.

Homeostatic plasticity is capable of developing therapeutically beneficial setpoints as well as pathologic setpoints.^3^ Because plasticity is largely experience-dependent, changes in the external environment can dramatically modify the activity of a neural network and its consequent attractor network. One example of this potential is a change in social network and support. Individuals experiencing poor quantity and quality of social interaction also report large deteriorations in mental health.^40^ On a circuit level, social isolation acts with circuits historically correlated with depression in a parallel manner.^40,41^ These data suggest that social isolation encourages a new homeostatic setpoint, altering neural network activity, and hyperstabilizing a pathological attractor state. With this new setpoint, acute improvements of one’s social network would do little to permanently modify the firing rate at a population level in these circuits. Thus, acute intervention with no chronic effect will not improve mental health long-term. With improvements in the quantity and quality of social stimuli on a chronic scale, a new homeostatic level of firing rate is established, thus shifting a pathological attractor state into a salubrious one.

## STIMULUS PROCESSING INVOLVES A BALANCE OF TOP-DOWN AND BOTTOM-UP

Changes in attractor state dynamics can result in major shifts in how stimuli are processed. At the algorithmic level, we distinguish between two feedback loops that, when balanced, allow for appropriate assessment and response to stimuli. The rapid and unidirectional BU feedback loop allows for immediate responses to sensory stimuli, whereas the slow and convoluted TD feedback loop allows for more complex assessments of the environment and fine-tuned behavior (Box 2). These loops proceed in three main steps: sensory detection, processing, and output (Figure 2A).

### Sensory detection

The first task of the system is to appropriately identify relevant information from an overwhelmingly stimulus-filled environment. *Input* signals, composed of both external (e.g., sounds from the environment) and internal cues (e.g., hunger), are detected by sensory processes in the body in a preattentive, automatic phase.^42,43^ Here, the brain must determine what is salient enough to bring to the forefront of attention and potentially act upon. For instance, studies have indicated that individuals have a latency bias toward identifying fearful stimuli among a background of nonfearful stimuli when compared to the reverse.^44^ Salient stimuli will meet an attention threshold at the first BU node: *association* (Figure 2A). Associative processes, both learned and innate, allow for valence assignment of the stimulus as positive (appetitive or rewarding), negative (threatening or aversive), or neutral.^45^ At the same time, input cues are sent to the central node of the TD loop: *cognition*, involved in conscious, goal-directed thinking.^42,46^

### Processing

*Integration* receives inputs from both BU and TD centers to contextualize and assess relevant information about the stimulus. The BU loop allows for information concerning the valence and intensity of the stimulus to be transmitted directly to the *arousal* node to bring the body into a state of psychological and physiological alertness. The TD loop may counteract these efforts by filtering out stimuli that are irrelevant or unnecessary for goal-oriented behavior.^47–49^ This allows for disengagement from the stimulus to prevent an inappropriate response. The TD loop continuously incorporates new information from changing environments and involves multiple iterative, inner feedback loops to continue amending cognitive assessments of stimuli. A loud barking sound, for example, might initially prompt a startle response, triggered by BU processing, but as more relevant information surrounding this sound is integrated by TD processes, one would notice the dog is safely held by an owner on the leash.

### Output and feedback

*Action selection* is again informed by a balanced interplay of both loops: arousal and cognition. Once the action has been selected, a *behavioral response* is produced, to move toward or away from an external stimulus (e.g., moving away from a spider) or to change an internal state (e.g., eating food). Action selection feeds back into the cognitive node allowing for future strategization and goal orientation. These actions reconfigure the set of stimuli presented to the system, completing the loop.

In a healthy state, these parallel processes allow for appropriate and evolutionarily adaptive behavioral responses that can change depending on the valence, intensity, and relevance of stimuli. The BU loop, driven by reflexive, innate, emotional responses to stimuli, allows for rapid, immediate processing to respond to very threatening or very rewarding stimuli (Figure 2). As stimuli valence intensifies (more positive or negative), arousal generally increases,^45^ resulting in a higher likelihood of a behavioral response. The TD loop prevents unnecessary increases in arousal levels by filtering out stimuli that are neutral and irrelevant (Figure 2A).^9^

## MALADAPTIVE STIMULUS PROCESSING AND VALENCE DESTABILIZATION RESULT IN DISEASE STATES

Although there is heightened negative affect and bias toward evaluating stimuli as negative in both anxiety and depression,^42,47,50^ the manifestation of these diseases is often quite different. For instance, the prospect of interacting with a peer may provoke negative nervous or threatening feelings for someone experiencing anxiety, whereas for someone with depression, the same prospect, though also negatively evaluated, might induce feelings of fatigue. In Clark and Watson’s tripartite model of anxiety and depression, negative affect is the common link between anxiety and depression, and contributes to anxious-depressed comorbid states.^50^ In such comorbid states, increased attractor state depth results in maladaptive behavior and increased negative affect. In this review, we focus on the phenotypes identified in the tripartite model that distinguish these two disease states: increased physiological arousal in anxiety, and anhedonia and disengagement from the environment in depression.^50^ We propose that an imbalance in the two processing modes leads to these distinct disease phenotypes.

As reviewed in detail by Mogg and Bradley, in an anxious state, the system shifts overwhelmingly toward BU processing, without enough regulation from TD, disrupting goal-oriented behavior.^51^ This leads to excessive physiological and psychological arousal and increased sensitivity to environmental stimuli, prompting stimuli to be biased as threatening^9,42,51,52^ (Figure 2B). Stimuli that would commonly be perceived as neutral might trigger psychological symptoms associated with anxiety, including hypervigilance and worry, as well as physiological symptoms, such as increased heart rate, sweating, and muscle tension (Box 1). In depression, TD cognitive processes take control over emotional, reflexive ones, and overinhibit the BU loop. This can lead to the inability to engage appropriately with the environment or reach the arousal threshold to act upon relevant stimuli (Figure 2C),^47,50,51^ manifesting as a lack of motivation and anhedonia (Box 1). Within this model, bidirectional overcompensation in individuals with comorbid anxious-depression leads to a maladaptive alternation between BU- and TD-biased processing (Figure 1D–F).

Human studies show that emotion and attention processing activate distinct patterns of activity in the brain depending upon whether a TD- or BU-eliciting stimulus is presented.^53,54^ BU-inducing stimuli, such as images that can quickly provoke an automatic emotional response, have been shown to activate brain regions, such as the amygdala, whereas TD-inducing stimuli (words and imaginative activities) have implicated the PFC.^53,54^ We focus on the amygdala and PFC as representatives of BU and TD processing, respectively, and elucidate how the biological mechanisms implement chronic changes on the computational and algorithmic level.

## ANXIETY DISORDERS CAN ORIGINATE FROM HYPERACTIVITY IN BOTTOM-UP REGIONS

BU processing at homeostatic levels is necessary for an animal to remain vigilant and cautious; however, pathological increases of activity in areas associated with adaptive anxiety processing can lead to maladaptive anxiety symptoms and behavior.^9^ Within the context of BU processing, the amygdala is a critical region in the brain for associative learning, BU processing, and arousal.^44,54–56^ The amygdala is also involved in processing changes in the internal state as an important interoception axis.^57,58^ What must be noted is the theorized bidirectional relationship between interoception and emotional arousal. As internal states change, emotions may arouse through conscious or subconscious evaluation of these homeostatic deviations.^59^ This interplay between internal state and emotional response reflects classic theories of emotion^59,60^ and illustrates how amygdala activity can simultaneously reflect interoceptive and emotional status.^54,58^ As evidenced by experiments in humans, patients with amygdalar insults struggle to generate physiological responses in anticipation of risky behavior or in response to losing in a gambling task.^61^ Further human behavior experiments show that associative processing related to emotion is also impaired—patients with amygdala lesions fail to recognize fearful emotions in faces, although they can decipher personal identity.^62^ Amygdala volume correlates positively with fearfulness in human subjects while conversely, bilateral damage to the amygdala is associated with decreased anxiety and fearfulness.^63^ The basolateral amygdala (BLA) specifically is a driving factor in anxiety,^64^ composing one-third of a tripartite group of regions with reciprocal connections that are heavily involved in emotional processing, including the ventral hippocampus, and the medial PFC (mPFC).^65–67^ We will focus specifically on the relationship between the mPFC and the BLA, due to their theorized roles in our algorithmic processing model and their respective involvement in emotional processing.

In human fMRI studies, the functional connectivity between the BLA and the mPFC has been correlated with higher levels of anxiety.^68,69^ Human fMRI investigations into different biotypes of mood disorders have elucidated that dysfunctional connectivity between the PFC and the amygdala increases anxiety.^70^ Rodent studies have specifically illustrated that the directionality between the BLA and the mPFC is relevant in mediating fear and anxiety-like behavior. From a processing perspective, amygdala projections to the mPFC encode motivational and arousal information, which can be modulated and filtered by the mPFC. These data are bolstered from rodent studies using imaging,^71^ electrophysiological,^72,73^ and optogenetic^66,74^ techniques, which have elucidated that BLA input to the mPFC is necessary for anxiety behaviors. Theta oscillations between the BLA and the mPFC synchronize in anxiogenic environments; oscillatory stimulation is sufficient to induce anxiety-like behaviors in rodents.^66,75,76^ Excitation of BLA projections to the mPFC produces anxiogenic effects, freezing responses, reduction in social interactions, and attenuation in cue-associated fear in mice; inhibition of BLA–mPFC projections facilitates social interaction and reduces freezing.^74,77^ The role of the BLA in fear learning fits the role of the association node, projecting rapid associative information to the integrative mPFC. Amygdala inputs drive feedforward inhibition of mPFC neurons by targeting parvalbumin (PV)-positive interneurons.^72^ These results suggest the associative information from the BLA impacts on neural ensembles in the mPFC, possibly by reducing background activity and allowing for appropriate neurons to integrate and direct behavior. When considering these results together, hyperexcitability in the BLA biases the BU pathway and represents an entrenched BU attractor state in anxiety disorders.

## DEPRESSION MAY EMERGE FROM HYPERACTIVITY IN TOP-DOWN REGIONS

The mPFC has been historically linked to mood disorders and connectivity within this region has been implicated in depression. Encoding in the mPFC is attributed to reward learning,^78^ emotional memory,^56^ decision making,^79^ and moral assessment.^80^ Patients with vmPFC lesions exhibit impaired strategic decision making^61^ and intuitive moral judgment,^81^ suggesting that the mPFC plays a critical role in TD processing.^82^ Activity in the frontal cortices, specifically the anterior cingulate cortex, is associated with increased optimism and likelihood to imagine positive future outcomes, suggesting that the mPFC is required for long-term mediation that is characteristic of TD processing.^83^ Experimental and functional imaging studies have demonstrated dramatic changes focused on the PFC in depression.^79,84,85^ MDD has been associated with hyperactivation of the mPFC and reduced activation in reward-processing dopaminergic neurons in the nucleus accumbens (NAc).^85–87^ Deep brain stimulation targeting the PFC, namely, the subgenual cingulate region, resulted in profound antidepressant effects; however, others have struggled to replicate these results.^88,89^ What is also of importance are the connections between the mPFC and other regions, namely, the ventral tegmental area (VTA) and lateral habenula (LHb).^90–93^ The VTA is highly associated with reward and motivated behavior, whereas chronic inhibition of VTA–dopamine (DA) neurons induces depressive-like symptoms.^94^ Activity in the VTA is also susceptible to stress on different timescales—acute stress increases VTA neurons projecting to the mPFC, while chronic stress depresses VTA–mPFC activity.^90,95^

The LHb also regulates reward-prediction responses and has recently been implicated in regulating negative associations.^92^ Because of this unique signaling pattern, the LHb has been referred to as the brain’s “anti-reward center” and receives input from a diverse set of neurotransmitters.^6,69^ Increasing excitability of LHb neurons via optogenetic^96^ and genetic^97^ methods is sufficient to induce avoidance behavior, anhedonia, and other core depressive symptoms^6,98^ in rodent models. In contrast, clinical studies applying deep brain stimulation to inhibit the LHb observed antidepressive effects.^99^ The LHb receives primarily glutamatergic inputs from mPFC.^92^ Following the upstream encoding of reward and cognition, the mPFC–LHb circuitry is likely critical for behavioral control over negative affectation. Research investigating the mPFC, LHb, and VTA suggests a relationship in which decreasing transmission from the VTA leads to an excitation/inhibition (E/I) imbalance in the mPFC, which is reflected as hyperexcitability in projecting regions like the LHb (Figure 3C). Interestingly, the LHb projects back to the VTA, potentially constructing a TD processing loop that is hyperactive in negatively biased valence disorders like depression.^99^

## OSCILLATORY ACTIVITY AS A DRIVER FOR TOP-DOWN/BOTTOM-UP BALANCE

Proper attention to stimuli relies on a healthy balance of TD and BU processing. In contrast, salient stimuli are processed in a BU fashion through sensory inputs, deliberate TD control of attention allows one to focus not only on vestibular senses but also on the long-term evaluation of these stimuli.^100,101^

Oscillatory activity in the brain refers to the rhythmic and synchronously phase-locked activity between brain regions.^102^ This synchrony allows for distant brain regions to communicate and hold long-term information for conscious problem-solving by linking the information in these regions via a temporal framework. In both humans and monkeys, local field potential (LFP) gamma frequency (25–140 Hz) coherence between the PFC and visual cortex drastically increases during attention to a visual stimulus, implicating a concerted role of the mPFC for driving long-term attention.^103^ This type of oscillatory activity also provides the PFC with the ability to represent a variety of distinct categories of information in concert, allowing for more complex working memory.^104,105^

Oscillatory synchrony between the amygdala and the mPFC may be critical for healthy emotional evaluation of stimuli and may help bias the brain into certain states. Theta (3–8 Hz) oscillations between these brain regions are enhanced during fear learning in both rodents and humans, and disruption of these theta rhythms affects fear extinction or recall depending on the theta phase.^106–108^ Similar frequencies between the PFC and the hippocampus regulate avoidance behaviors in rodents, and phase-locked stimulation at these frequencies also enhances avoidance.^109,110^ These data imply that when signals are in phase, synchronous activity is amplified to bind certain inputs together and encourage attractor state transitions. Additionally, if these oscillatory dynamics are disrupted, it may lead to maladaptive emotional processing and brain states. For example, asymmetric alpha (8–12 Hz) activity between the left and right prefrontal regions has been commonly found in patients with MDD.^111–113^ Reduced gamma and alpha activity is also found in patients with euthymic bipolar disorder, characterized by a lack of mood disturbances, suggesting a shift in oscillatory brain states during certain emotional phases.^114,115^ Enhancing certain rhythms may also aid in the transitions to healthier attractor states. Disrupting the mPFC through stress can lead to the reduced amygdala and VTA synchrony and increase emotional pathologies found in anxiety and depression.^116^ Recently, work has been done to map how oscillatory activity propagates throughout the brain rather than through a simple pair-wise comparison between two brain regions. The collection of factors (spectral power, synchrony, and phase directionality) as observed through LFPs comprises the conceptual “electome” framework. These electome factors describe how oscillatory activity may shift brain dynamics into system-wide states and how certain states have been shown to predict vulnerability to depression.^117^ These data suggest that oscillatory synchrony may be integral for healthy brain states, and disruption of this synchrony may lead to unstable transitions between these states.

Dysfunctions in oscillatory activity may lead to maladaptive transitional patterns between these states, leading to conditions that may either be stuck in one attractor versus the other, or with very shallow attractors enabling high transition probability manifesting as comorbidity with anxiety-related and depressive symptoms. As oscillatory activity in attention suggests an upper limit to how much information the brain can attend to at one time;^104,105^ such may be the case when patients experience symptoms related to both anxiety and depression. It may be difficult to imagine feeling both anxious and apathetic to a stimulus at once, but imbalanced oscillatory activity may lead to rapid transitions between these states, which is perceived as these symptoms being experienced at the same time in comorbidity.

## NEUROTRANSMITTER BALANCES DRIVE ATTRACTOR AND MENTAL STATE STABILITY

Glutamate and gamma-aminobutyric acid (GABA) are the primary neurotransmitters involved in the excitation and inhibition of activity, respectively. Metabolically, glutamate acts as a precursor for GABA, which contributes greatly to controlling neural network dynamics through inhibitory action.^118,119^ GABAergic interneurons can be identified by their expression of somatostatin (SST) and PV, among other markers. Postmortem studies of patients with depression have shown a reduction of SST/PV interneurons in the PFC.^120,121^ Treatments with various antidepressants, electroconvulsive therapy, and cognitive behavioral therapy restore GABA levels in depressed subjects.^122^

While glutamate and GABA drive excitation and inhibition post-synaptically, they also contribute to and are subjected to changes in plasticity. GABAergic transmission in the mPFC constrains LTP, depending on the presence of either GABA-A or GABA-B receptors.^123,124^ GABA-A receptor (GABA-AR) mutant mice have significantly less surface expression of NMDAR and AMPARs and exhibit anhedonia and behavioral inhibition.^125,126^ Chemogenetic inhibition of GABAergic neurons in the mPFC is not only sufficient but necessary for antidepressant responses.^127^ Because GABAergic interneurons are responsible for controlling E/I within the cortex and excitatory projections, hyperactivation of GABAergic interneurons supports a model in which E/I imbalances cause anhedonia and other depressive symptoms^119,128,129^ (Figure 3C).

The E/I balance in the mPFC can be disrupted by a dearth of inhibitory GABAergic neurons. Our model hypothesizes that unregulated excitatory activity in the mPFC can lead to excitotoxicity in the region (leading to observed structural changes) and hyperexcitability in downstream projections like the LHb causing aversive behaviors.^92^ This hypothesis is further supported by human imaging studies that demonstrate a biotype of depression in which frontostriatal networks have pronounced hyperconnectivity in patients with more severe anhedonia and motor retardation.^70^ The consequences of this regional specificity are reflected in the differential effects of anxiety and depression drugs, specifically ketamine; recent studies have demonstrated that ketamine preferentially increases LTP in GABAergic interneurons in the mPFC, potentially rectifying the E/I imbalance.^130^ Glutamate causes the excitation of neurons, and excessive glutamate may drive excitotoxicity in the mPFC in the case of depression.^124,131,132^ Patients with MDD and postpartum depression have marked elevations of glutamate in the mPFC.^124,132^ Reciprocally, increased glutamatergic signaling from the BLA to the mPFC causes anxiety-like behavior. Glutamate intake at excitatory synapses can attune the polarity and magnitude of long-term plasticity changes, demonstrating that chronic increases in glutamate release can manipulate the basal setpoint of circuits, leading to hyperreactive neuronal populations.^133^ In both anxiety and depression, human and rodent studies demonstrate that increased glutamate is present in either the BLA or the mPFC, respectively. These tonic changes in glutamate can represent deepened attractor states and, thus, one can use neurotransmitter levels to model TD- and BU-biased processing modalities.

## NEUROMODULATION IN TOP-DOWN AND BOTTOM-UP PROCESSING CIRCUITS INFLUENCES PLASTICITY AND MENTAL STATE

Fast ionic signals represent the movement of the brain state, but to shape the manifold upon which our activity travels, the brain must operate on other timescales, including using long-term affective signaling like neuromodulation. While an animal acutely responds to stimuli, the strategies by which it responds, and the lasting effect of cumulative stimuli must have a longer-term effect. The mood reflects the summative impact of expectations and reward outcomes.^20^ The metaphorical narrative surrounding mood is akin to the geometry of attractor state landscapes.^134^ Where the processing is how the ball moves across energy landscapes, mood describes the energetic limitations of the said landscape, or the dimensions of an attractor state. Experiences affect mood, which in turn is integrated to affect subsequent experience through neuromodulation and plasticity alterations.

There is a strong relationship between the level of expression of given neuromodulators and mental state. Neuromodulator release in the brain has been linked to anxiety and depression and provides insight into how mental illness is encoded.^127^ As computational theorists of attractor states argue, the stability of an attractor state is analogous to the level of plasticity in the region. Thus, the presence and identity of neuromodulators inform our model through an explanation of how selective changes in plasticity alter attractor state stability. We will be specifically focusing on the role of serotonin (5-HT), norepinephrine (NE), and DA, and their role in plasticity in TD and BU regions. We suggest a model in which regional dysfunctions of modulation drive anxiety and depression symptoms, entrenching hyperstable attractor states. In our model, we argue that anxiety is caused by a hyperstable attractor state entrenching BU-biased processing.^135^ This state is due to the hyperexcitability of the BLA and its glutamatergic projections, namely to the mPFC.^72^ Conversely, depression is caused by a hyperstable TD-biased attractor state landscape, in which an E/I imbalance is maintained by disordered neuromodulation in the mPFC.

## DISORDERED NEUROMODULATION: SEROTONIN

5-HT has been historically linked to emotional behaviors, with a likely role in TD/BU processing motifs. In the amygdala, infusions of serotonin influence fear learning in mice. Microdialysis studies demonstrate that inescapable shock enhances 5-HT levels in the BLA from both conditioned stimulus and unconditioned stimulus presentations.^136^ Further investigations on fear learning demonstrate that 5-HT signaling is positively correlated with fear memories. Mice that overexpress 5-HT transporter (5-HTT), a protein that clears 5–HT from the extracellular space, demonstrate impaired fear learning, whereas the 5-HTT–underexpressed counterparts have impaired fear extinction. In primates, manipulations of 5-HT receptors affect trait anxiety-like phenotypes.^137^

The role of 5-HT in fear learning in the amygdala supports the hypothesis that 5-HT causes changes in plasticity. 5-HT release in the BLA depresses excitatory postsynaptic currents from outside inputs, likely via inhibitory GABAergic neurons.^138,139^ Simultaneously, the application of 5-HT increases the excitability of BLA pyramidal neurons—suggesting that 5-HT release increases excitability within the BLA.^140^ Activation of 5-HT_2A_ receptors (5-HT_2A_Rs) enhances NMDAR-mediated potentials, demonstrating that 5-HT_2A_Rs facilitate NMDAR-dependent synaptic plasticity in the BLA.^141^ This model has been fortified by results demonstrating that chronic administration of selective serotonin reuptake inhibitors (SSRIs; e.g., fluoxetine) increases plasticity by decreasing the number of PV+ interneurons in the BLA.^138,141^ Establishing a 5-HT–induced hyperplastic state in the amygdala led to the elimination of fear memories—suggesting that 5-HT likely influences excitability in the amygdala, and increases in 5-HT or 5-HT-receptor sensitivity modulate anxiety states and likely emotional processing in general.^136,140–142^ In our TD/BU processing model, increases in amygdala sensitivity cause a BU processing bias. Our model would thus hypothesize that increases in 5-HT levels would cause a BU processing bias and increase spontaneous emotional processing. Indeed, SSRIs have historically been used to treat disorders of negative affect, including depression and anxiety.^143^ In both healthy and depressed individuals, chronic SSRI administration leads to a positive shift in valence processing, possibly via amygdalar circuits.^139,144^ Further studies investigating SSRI administration demonstrate that SSRI treatments (increasing levels of 5-HT in the synaptic cleft) enhance LTP from BU regions communicating to the mPFC.^141^ These results suggest that excess 5-HT signaling acutely mediates plasticity in mPFC.

While 5-HT facilitates excitability in the BLA, the mPFC both receives 5-HT inputs and exerts TD control of 5-HT expression in downstream regions. 5-HTergic inputs from the mPFC are complicated by the heterogeneous encoding of different 5-HT receptors.^140^ Human blood-oxygen-level-dependent (BOLD) fMRI studies reveal a positive relationship between 5-HT_2A_ receptor activity and amygdala reactivity to anxious stimuli, which is inversely moderated by 5-HT_1A_ receptor activity.^145,146^ 5-HT_1A_ and 5-HT_2A_ receptors are localized in a relevant orientation–the inhibitory 5-HT_1A_ receptor is located on the axon hillock, whereas the excitatory 5-HT_2A_ receptors are located on the dendrites of glutamatergic neurons.^145^ Given this orientation, 5-HT_1A_ receptors can effectively gate 5-HT_2A_ receptor activity and PFC output, suggesting that 5-HT signaling in part modulates TD control from the mPFC to the amygdala.^147^ Thus, disordered localizations of 5-HTergic signaling could deleteriously influence the inhibitory brakes on fast, associative BU processing.

Studies investigating the dynamic changes in 5-HT signaling in the mPFC provide more insight into how 5-HT can mediate TD processing and plasticity. 5-HT_2A_ receptor activation enhances NMDAR-dependent plasticity, strengthening associative memory.^141^ Rodent studies comparing responses to stressful experiences demonstrate that 5-HT sensitivity significantly increases as a response to stress in the mPFC, and high increases in DA efflux follow.^136^ Interestingly, 5-HT_1A_ agonism decreases both 5-HT and DA release, further suggesting that low 5-HT_1A_ receptor binding is required for TD mPFC processing.^145^ Whole-life knockout of 5-HT_1A_ receptors in mice leads to a depression phenotype, suggesting that without the needed gating of 5-HT_2A_ signaling, the mPFC exerts excessive TD control, leading to a phenotype that is hyporesponsive to stress.^148,149^

The relationship between 5-HT and DA signaling is supported by developmental studies, which show that lesioning of 5-HTergic projections to the mPFC increases DA release.^150^ These data and the aforementioned receptor data together suggest a model for 5-HT control of stress responses in the amygdala which projects excitatory input to the mPFC. Agonism of 5-HT_1A_ receptors enhances DA signaling in the mPFC, suggesting a broader involvement between 5-HT and DA release.^151^ 5-HT signaling likely encompasses a modulatory role in the mPFC, flattening attractor state transition probabilities and selectively altering plasticity, depending on the activated receptor (Figure 3A). Disordered localization of the 5-HT signal could disrupt this system and lead to pathological entrenchment of mental states like anxiety or depression.

## DISORDERED NEUROMODULATION: DOPAMINE

Whereas 5-HT could flatten attractor state landscapes, DA signaling plays a role in the entrenchment of salient behaviors. DA computationally controls behavior in the mPFC by gating sensory input, manipulating memories, and relaying motor commands.^152,153^ This gating of activity has led to models that also suggest that DA modulates response to the saliency of reward.^154,155^ DA release has been theorized to reflect reward prediction errors (RPEs) in the mPFC.^153^ Inactivation of the mPFC in classical conditioning tasks with a differing probability of reward affects DAergic signaling when the reward is not guaranteed.^78^ DA signaling in the NAc core does conflict with RPE, as experimental results cannot replicate RPE in practice.^155^ Dysfunctional DA activity is correlated with problems in processing motivation, pleasure, and reward.^95,152,156^ Imaging studies in patients with social anxiety reflect a positive correlation between symptom severity and DA transporter proteins (DAT) availability in the amygdala and hippocampus.^157,158^ Moreover, 5-HT and DAT coexpression was significantly increased in the amygdala of patients with anxiety, suggesting that DA may also play a role in the progression of anxiety.

Patients with depression report difficulties with motivation (anhedonia) and reward processing, both of which DA is intimately involved in encoding in the mPFC. What is of relevance is the relationship between the mPFC and the VTA, a circuit that is historically linked to depression.^95,159^ Phasic stimulation of VTA-DA inputs to the mPFC also increases conditioned place preference.^160^ Alongside evidence that VTA-DA neurons lack activity in patients with depression, these data support a model in which a dearth of DA input to the mPFC enhances depressive symptoms. In mice with chronic stress-induced anhedonia, decreased levels of DA release have been measured from the VTA projections to the mPFC. In social defeat stress models of depression, mice initially exhibit a hyperexcitability of VTA-DA neurons, as compared to resilient mice that exhibit stable firing.^95^ What provides more insight into how DA signaling influences mPFC activity is the role of DA inputs in plasticity. Experimental enhancement of VTA-DA neuron excitability achieved an antidepressant effect through homeostatic plasticity.^95^ These results support a model in which homeostatic plasticity interacts with DA neurons in the brain to modulate depression states. Anxiety circuits can be similarly altered via chronic stimulation, altering excitability levels to a healthier homeostatic setpoint.^161^

DA exerts influence through D1-like (type 1/5) and D2-like (type 2–4) receptors, which causes increases and decreases in levels of the messenger molecule cyclic adenosine monophosphate (cAMP), respectively.^162,163^ Changes in cAMP retroactively recruit or inhibit NMDAR signaling; thus, DA signaling can bidirectionally alter plasticity. DA release, therefore, plays an important role in the modulation of GABA/glutamate signaling in the mPFC.^163^ Inactivation of DA release is sufficient to eliminate reinforcement learning behaviors normally attributed to GABA/glutamate signaling.^163^ These data ultimately support a theory of regional-specific alterations in DA signaling in the states of depression and anxiety.

5-HT and DA both mediate NMDAR recruitment and LTP in the amygdala, encouraging greater sensitivity in the BLA. Behaviorally, D1 agonism in the amygdala elicits anxiogenic effects, whereas antagonism elicits anxiolytic effects.^164,165^ D2 agonism/antagonism illustrates more mixed results, suggesting that D1 receptors in the BLA encode rapid associative processing in the BLA, whereas D2 signaling develops adaptive responses. D1 receptor activation in the BLA further dampens mPFC-induced inhibition, suggesting that the mPFC regulation of BLA activity is inversely dependent on DA levels in the BLA.^165,166^ These data suggest that excess D1 signaling could drive BLA excitability and, given the role of D1 receptors in increasing NMDAR conductance, deepens a BU-biased attractor state landscape.

The contributions of DA signaling in the mPFC are necessary for motivation and emotional processing; thus, our model attributes a lack of DA signaling to an imbalance between excitatory and inhibitory neurons in the mPFC, leading to anhedonia.^167^ DAergic control of the E/I balance in the mPFC is influenced upstream by 5-HTergic signaling, possibly causing an increase in inhibitory signaling and plasticity in the mPFC.^167,168^ Whereas 5-HTergic signaling seems to flatten attractor state landscapes, DAergic signaling defines and entrenches them.^12^ Recent rodent studies have shown that DA-dependent plasticity is occluded in the mPFC of rats susceptible to chronic mild stress, although this effect is reversed following the administration of ketamine.^167^ Following our tri-level model, these DAergic data suggest that disordered DA inputs to the mPFC would deleteriously increase the depth of a point attractor state, leading to excess TD control over other regions and depressive symptoms.

## DISORDERED NEUROMODULATION: NOREPINEPHRINE

Functional DAergic signaling in the mPFC is not possible without intact NE release.^169^ DA and NE interact to regulate excitatory and inhibitory firing in the mPFC and maintain a delicate balance; too little DA/NE influence leads to memory impairment, whereas excess agonism of DA/NE receptors leads to excitotoxicity and dysfunction.^169,170^ Much like DA, NE is released in the amygdala and the mPFC in response to acute stimuli like anxiety or reward and is involved in sleep/wake cycling, stress, and fear responses^171^ (Figure 3B). Due to its involvement in processes that are so often disordered in anxiety and depression, human studies have found correlative links between disordered NE release and mental illness. NE transporter and 5-HTT polymorphisms are related to increased susceptibility for anxiety in human subjects.^137,172,173^ NE neuronal cell groups can activate the hypothalamic–pituitary–adrenal axis in stress responses, consolidate negative emotional memory through amygdalar and hippocampal projections, and control reward evaluation in the PFC.^174,175^ In the cortex, NE inhibits cAMP, prolonging potassium currents and stabilizing attractor state networks.^176^ In the amygdala, the release of NE has a similar role to 5-HT in regulating brain-derived-neurotrophic-factor-positive neurons, suggesting that it strengthens synaptic plasticity in BU processing regions as well.^177^ While NE release is necessary for processing reward in the mPFC, continuous NE release from chronic stress causes deleterious effects in cortical processing.

DA and NE affect the behavior of E/I neurons by reducing feedforward GABA inhibition and enhancing LTP.^119^ Increased levels of DAT and NE in patients with anxiety disorders bolster this interpretation that excess monoamine release causes downstream dysregulation of GABA.^158^ Injection of corticosterone into the amygdala induces anxiety-like behavior, imitating depressed GABAergic tone in the lateral amygdala.^121^ In addition to inducing a hypervigilant state, corticosterone increases the release of DA^178^ and NE^179,180^ in BU regions. Bidirectionally, the inhibition of GABA pyramidal neurons in the BLA promotes fear learning.^181^ Because GABAergic interneurons innervate and modulate glutamatergic outputs from the amygdala, reduction of GABA release triggered by dysfunctional neuromodulation may cause hyperexcitability of glutamatergic neurons.

Chronic release of NE via repeated traumatic stress can cause the consolidation of a lasting negative affective bias.^182^ Human subjects describe acute systemic infusions of NE as hyperarousal and later describe a “numbing” experience (when circulating NE is much lower, potentially representing NE “burnout”)^175^ (Figure 3B). As NE and 5-HT interact in anxiety, they also have a relationship in depression. When drug-naïve patients with depression were treated with an NE reuptake inhibitor (NRI) versus an SSRI, patients given SSRIs experienced a relapse of symptoms, whereas NRI patients did not.^183^ These data together with data demonstrating the role of NE in plasticity suggest a powerful effect of dysfunctional NE release, which acutely deepens attractor states. Following behavioral data in humans and rodents, it is possible that excessive NE release due to chronic stress could promote dysfunction in the mPFC and the amygdala, leading to an anxious-depressive model with two hyperstable attractor states.^25^ This model predicts behaviors that are typically seen in cases of unstable NE, DA, and 5-HT firing, including impairments in cognitive flexibility, working memory, hypervigilance, and negative valence bias. Recent research into the role of neurotensin demonstrates that it encodes valence assignment in the BLA by exerting a modular influence over synaptic plasticity in a valence-dependent manner.^184^ Given that patients with anxiety and depression suffer from negative valence bias, neurotensin signaling may be impaired in these mood disorders.^184,185^ Reciprocally, neurotensin inhibits DA-mediated suppression of VTA neurons,^186^ suggesting that disordered neurotensin modulation could impact VTA–DA signaling to the mPFC, causing disordered DAergic firing.

## CONCLUDING REMARKS AND FUTURE PERSPECTIVES

While attractor state modeling argues how anxious and depressive states persist, the question still stands: how do these disease states develop, and what causes a transition into these states? Modeling of other natural phenomena, such as earthquakes, nuclear chain reactions, forest fires, and avalanches, all exhibit activity that can be described by power laws; neural activity can be modeled the same way.^187,188^ Neuronal processing requires the integration and redistribution of thousands of inputs with dynamics described as neuronal avalanches.^187^ This theory suggests that as a population nears a critical threshold, the observable behavior does not change until one unit exceeds threshold and causes many other units to do so in turn.^189^ This dynamism is described in the critical brain hypothesis, which suggests that neuron populations operate in the vicinity of the critical point of a phase transition, allowing for variable dynamics (through neuronal avalanches) from rest.^190^ While attractor states explain the stability and perpetuation of a given state, neuronal avalanches and the broader critical brain hypothesis explain the passage from one attractor state to another. In a healthy system, neuronal avalanches can elicit state transitions; however, in an unhealthy system, neuron populations would struggle to reach the criticality necessary for state transition. Neuronal avalanches have been observed in cortical populations using *ex vivo* microelectrode array recordings^187^ and *in vivo* electrode recordings;^189^ suggesting a role of brain criticality in TD processing, but it has yet to be investigated if neuronal avalanches are observed in subcortical regions.

As a class, mental health disorders present the largest economic burden to our society as well as being the least well-understood in terms of biological mechanisms. Many disparate subfields interface with mental health disorders, from computational psychology to neuropharmacology to modern circuit neuroscience, yet they are poorly integrated. Here, we have linked the computational, algorithmic, and implementational levels of investigation to connect the conceptual frameworks posited by each field. On a computational level, the function of the mind in health and disease can be compared to attractor states, analogous to brain states. On an algorithmic level, we explore how TD or BU brain states represent psychological processing pathways that are mediated by neural circuits. On an implementational level, we can probe the way synaptic changes can alter neural dynamics and shift brain states along multiple distinct parameters. Our review presents a framework through which to understand and decipher the differences observed in anxious and depressed subjects based on behavioral and neural readouts. Future studies should test the idea of a shift in processing modalities in more depth by directly measuring and comparing neural processes implicated in these two modes of anxious and depressed individuals while responding to various stimuli. Additionally, computational analyses of brain states in animal models may reveal the attractor networks mediating anxiety and depression.

To forge a path forward amidst the last frontier of our understanding—ourselves—we must integrate and synthesize the diverse perspectives for investigating psychiatric disease. This conceptual model is largely speculative and subject to evolution. Directed and focused investigation along multiple levels will enable the diagnosis of brain-based diseases using both behavior and brain activity as readouts.

## BOX 1

**Table:**

Generalized Anxiety Disorder (GAD)	Major Depressive Disorder (MDD)
**Physiological:** Restlessness, fatigue, muscle tension, sleep disturbances, irritability, and hypervigilance. Symptoms are unrelated to the effects of a substance or medical condition. **Psychosocial:** Persisting excessive apprehension, expectation of danger, and worry about a number of life events or activities.	**Physiological:** Diminished interest and pleasure (anhedonia). Low energy, or a decrease in efficiency to complete routine tasks. Significant changes in appetite. **Psychosocial:** A sense of worthlessness or excessive guilt. Impaired ability to concentrate or make a decision (rumination). Recurrent suicidal ideation, or suicide attempts.

## BOX 2

**Table:**

***Bottom-up*** is the initial, rapid mode of processing that relies heavily on an immediate, less conscious, and more emotional reaction to salient stimuli from the environment. This type of processing is adaptive, allowing these to be rapidly and easily identifiable (e.g., being startled by a dog barking)	***Top-down*** is a slow mode of processing that relies on a cognitive, context, prior-knowledge, and goal-driven approach to assessing stimuli. This type of processing acts as a filter to identify which of the initially perceived salient stimuli are relevant and deserving of attention and behavioral responses (e.g., recognizing the dog is behind a fence)

## OUTSTANDING QUESTIONS

- What happens on a chronic scale when you have a hyperstable state? Why are changes in homeostatic or metaplasticity not rebalancing attractor state geometry?
- How do TD and BU attractor states interact with each other? Do these attractor states represent a part of a greater energy landscape in the brain, and what shape does that broader landscape take in health and disease?
- How can we leverage our understanding of attractor states to develop individual treatments and/or predict disease biotypes?
- How can we better model comorbid types of anxious-depression to further investigate neuromodulatory influence and plasticity change?
- Are there shared circuits or a divergent point in processing that account for negative affective states in both anxiety and depression?
- Major life changes in specific developmental periods make individuals highly susceptible for the development of mental illness. How does stress in specific developmental periods (e.g., childhood, adolescence) uniquely alter plasticity in cortical and limbic regions?
- Are the changes in plasticity that we see in patients with anxiety and depression indicative of a genetic susceptibility to these illnesses?
